# Cytological patterns of bronchoalveolar lavage fluid in mechanically ventilated COVID‐19 patients on extracorporeal membrane oxygenation

**DOI:** 10.1111/crj.13481

**Published:** 2022-03-11

**Authors:** Sebastian Voicu, Isabelle Malissin, Adrien Pepin‐Lehaleur, Laetitia Sutterlin, Giulia Naim, Aymen M'Rad, Emmanuelle Guerin, Jean‐Michel Ekherian, Nicolas Deye, Homa Adle‐Biassette, Bruno Mégarbane

**Affiliations:** ^1^ Réanimation Médicale et Toxicologique, INSERM UMRS1144, AP‐HP, Hôpital Lariboisière Université de Paris Paris France; ^2^ Service d'Anatomie et Cytologie Pathologiques, INSERM UMR 1141, AP‐HP, Hôpital Lariboisière Université de Paris Paris France

**Keywords:** ARDS, bronchoalveolar lavage, COVID‐19, ECMO, lymphocyte, neutrophil

## Abstract

**Introduction:**

Coronavirus disease‐2019 (COVID‐19) may lead to acute respiratory distress syndrome requiring extracorporeal membrane oxygenation (ECMO). Patterns of inflammatory bronchoalveolar cells in COVID‐19 patients treated with ECMO are not well described.

**Objective:**

We aimed to describe inflammatory cell subpopulations in blood and bronchoalveolar lavages (BALs) obtained in critically ill COVID‐19 patients shortly after ECMO implementation.

**Methods:**

BAL was performed in the middle lobe in 12 consecutive ECMO‐treated COVID‐19 patients. Trained cytologists analyzed peripheral blood and BAL cells using flow cytometry and routine staining, respectively. Data were interpreted in relation to dexamethasone administration and weaning from ECMO and ventilator.

**Results:**

High neutrophil proportions (66% to 88% of total cells) were observed in the absence of bacterial superinfection and more frequently in dexamethasone‐free patients (83% [82–85] vs. 29% [8–68], *P* = 0.006), suggesting that viral infection could be responsible of predominantly neutrophilic lung inflammation. Successful weaning from ECMO/ventilator could not be predicted by the peripheral white blood and BAL cell pattern.

**Conclusion:**

High neutrophil proportions can be observed in critically ill COVID‐19 patients despite the lack of microbiological evidence on BAL of bacterial superinfection. Dexamethasone was associated with lower neutrophil proportions in BAL. Our study was probably underpowered to provide BAL cell pattern helpful to predict weaning from ECMO/ventilator.

## Graphical abstract

In critically ill COVID‐19 patients treated with ECMO, a high neutrophil proportion in bronchoalveolar lavage fluid is possible despite the absence of evidence for bacterial infection. Neutrophil proportion is significantly lower in patients treated with dexamethasone at the time of bronchoalveolar lavage.

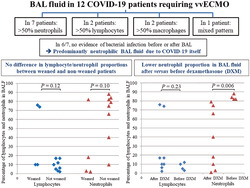

## 1 INTRODUCTION

Acute respiratory distress syndrome (ARDS) is a life‐threatening complication of coronavirus disease‐2019 (COVID‐19) pneumonia, which may require veno‐venous extracorporeal membrane oxygenation (ECMO) in addition to optimized mechanical ventilation.^1^, ^2^ Dexamethasone was shown to reduce 28‐day mortality among mechanically ventilated COVID‐19 patients.^3^ Therefore, investigating inflammatory cells in blood and lungs appears paramount to understand the overall process involved in the disease progression and how it is altered by steroid treatment.^4^, ^5^

Bronchoalveolar lavage (BAL) cells in COVID‐19‐attributed pneumonia are relatively well described.^6^, ^7^, ^8^, ^9^ However, description of BAL cell patterns and alterations resulting from dexamethasone administration in patients on ECMO remain scarce. Therefore, we designed this observational exploratory study (i) to describe inflammatory cell subpopulations in blood and BALs obtained in COVID‐19 patients shortly after ECMO implementation and as far as possible (ii) to seek any pattern associated with the successful weaning from ECMO and ventilator.

## 2 METHODS

All consecutive ECMO‐treated COVID‐19 patients with BAL admitted to our intensive care unit from March to May 2020 were included. ECMO was implemented in patients with refractory hypoxemia and/or hypercapnia despite muscular paralysis, optimized ventilation, and prone position, as recommended.^2^ Ventilator and ECMO settings were adjusted to maintain PaO_2_ ≥ 60 mmHg and sweep adapted to keep PaCO_2_ in the normal range if possible. Dexamethasone was administered as 10 mg twice daily for 5 days followed by 10 mg daily for 5 days,^10^ a higher dose regimen used before the Recovery trial.^3^ This study was part of the COVID‐ICU and French COVID‐19 cohort registries and approved by our institutional ethics committee (IDRCB, 2020‐A00256‐33; CPP, 11‐2020.02.04.68737).

BAL was performed in the middle lobe using ≥120 ml saline by trained pulmonologists and intensivists. Smears were analyzed microscopically after Papanicolaou, May‐Grünewald Giemsa, Ziehl, Grocott, and Perls staining by one experienced pathologist blinded to the patient treatments. Cell count in BAL fluid (BALF) was expressed as percentages of the total calculated on a 300‐cell sample. Tracheal aspirates were sampled every 24 or 48 h to detect bacterial infection. Within 24 h of BAL, peripheral white blood cells counts and CD3+, CD4+, CD8+, CD19+, CD16+, and CD56+ lymphocyte subsets were quantified using FACS‐Canto™ flow cytometry (Becton Dickinson, Erembodegem, Belgium). Clinical and routine biological data were collected from the patients' records.

Results are presented as median [percentiles 25th–75th] for the quantitative parameters and percentages for categorical variables. Data were compared using Mann–Whitney and Fischer's exact tests, as appropriate. *P* values <0.05 were considered significant.

## 3 RESULTS

Twelve out of the 17 ECMO‐treated COVID‐19 patients received BAL and were included (Table 1). ECMO was initiated 5 days [3–6] posttracheal intubation. PaO_2_/FiO_2_ ratio at ECMO initiation was 54 mmHg [48–61]. Patients received vasopressors (83%), inhaled nitric oxide (83%), almitrine (83%), and renal replacement therapy (75%). Hydroxychloroquine/azithromycin was administered in 83% of the patients. Eleven patients (92%) received dexamethasone. Duration of ECMO treatment was 20 days [18–33]. Four patients (33%) were successfully weaned from ECMO/ventilator and discharged alive from hospital whereas the rest died including three patients after ECMO weaning.

**TABLE 1:** Clinical characteristics and biological parameters in 12 critically ill COVID‐19 patients treated with optimized mechanical ventilation and veno‐venous extracorporeal membrane oxygenation

	All patients (*N* = 12)	Patients weaned from ECMO/ventilator (*N* = 4)	Patients not weaned from ECMO/ventilator (*N* = 8)	*P* value
Age (years)	56 [48–58]	49 [40–56]	58 [53–59]	0.09
Male gender, *N* (%)	11 (92)	4 (100)	7 (88%)	0.39
Past hypertension, *N* (%)	3 (25)	1 (25)	2 (25)	1.0
Diabetes, *N* (%)	2 (17)	1 (25)	1 (13)	0.58
Body mass index (kg/m^2^)	31 [27–34]	31 [29–34]	31 [27–34]	0.83
First symptom to ECMO (days)	13 [11–15]	14 [13–18]	12 [10–18]	0.39
Intubation to ECMO (days)	5 [2–6]	4 [2–4]	6 [3–7]	0.23
Intubation to BAL (days)	8 [5–16]	6 [4–7]	15 [7–21]	0.23
SOFA score on admission	10 [8–13]	10 [9–11]	11 [8–13]	0.67
Highest SOFA score	18 [15–19]	15 [13–18]	18 [17–19]	0.28
Dexamethasone treatment, *N* (%)	11 (92)	4 (100)	7 (88)	0.46
ECMO duration (days)	18 [14–26]	28 [14–47]	18 [14–22]	0.40
Mechanical ventilation duration (days)	25 [19–34]	47 [19–73]	25 [19–32]	0.55
Length of ICU stay (days)	33 [24–50]	90 [64–104]	29 [24–34]	0.13

Biological parameters at ECMO initiation				
PaO_2_/FiO_2_ ratio (mmHg)	54 [48–61]	49 [43–54]	59 [49–65]	0.13
Serum creatinine (μmol/L)	220 [85–332]	209 [70–344]	220 [128–294]	0.83
Serum alanine aminotransferase (IU/L)	31 [24–57]	30 [26–48]	40 [24–57]	0.83
Serum bilirubin (μmol/L)	15 [7–19]	11 [7–15]	17 [9–30]	0.28
Hemoglobin g/L	85 [77–10]	91 [77–11]	85 [76–89]	0.67
C‐reactive protein (mg/L)	94 [56–212]	134 [35–235]	94 [76–156]	0.67
Procalcitonin (μg/L)	2.4 [1.5–2.9]	2.2 [1.2–2.9]	2.4 [1.7–2.7]	0.83
Interleukin‐6 (pg/ml)	504 [49–1674]	551 [518–1077]	79 [15–1566]	0.29
Fibrinogen (g/L)	4.1 [3.1–6.8]	4.9 [2.2–7.9]	4.1 [3.7–6.1]	1.0
D‐dimer (ng/ml)	2555 [1488–5263]	1990 [1683–4343]	3175 [1770–5263]	0.67

*Note*: Data are expressed as median [percentiles 25th–75th] or percentages. Comparisons were performed using Mann–Whitney or *χ* ^2^ tests as appropriate.

Abbreviations: BAL, bronchoalveolar lavage; ECMO, extracorporeal membrane oxygenation; SOFA score, Sepsis‐Related Organ Failure Assessment score.

BAL was performed 5 days [2–10] post‐ECMO initiation under probabilistic wide‐spectrum antibiotics in all patients, including four dexamethasone‐free patients (i.e., three patients before dexamethasone initiation and the one who refused dexamethasone and eventually died) and eight patients on dexamethasone started 6 days [3–11] before. BALF analysis showed >50% macrophages in two patients (72% and 80%), >50% lymphocytes in two patients (74% and 76%), and >50% neutrophils in seven patients (66% to 88% of total cells; Table 2). Six patients among the seven had sterile tracheal aspirates before and after the BAL and were considered free of bacterial respiratory infections (Table 2). One patient had mixed cell populations with 43% macrophages, 40% neutrophils, and 17% lymphocytes. None of the patients had significant intra‐alveolar hemorrhage (maximum Golde score of 30 [score range, 0–400; normal <20; intra‐alveolar hemorrhage, >40]). Eosinophils and mastocytes were found in one patient, each at ~1% of the cells. No pathogen was documented in any of the patients on microscopic examination and culture.

**TABLE 2:** Ventilation and ECMO parameters, bronchoalveolar lavage fluid, and blood leukocyte subpopulations in 12 critically ill COVID‐19 patients treated with optimized mechanical ventilation and veno‐venous extracorporeal membrane oxygenation

	All patients (*N* = 12)	Patients weaned from ECMO/ventilator (*N* = 4)	Patients not weaned (*N* = 8)	*P* value
Ventilation and ECMO parameters at BAL				
Inspired O_2_ fraction (%)	100 [80–100]	90 [80–100]	100 [73–100]	1.0
Inspired tidal volume (ml)	231 [201–261]	216 [155–250]	240 [211–261]	0.67
Plateau pressure (cmH_2_O)	26 [24–27]	25 [23–26]	26 [26–28]	0.23
Static compliance (ml/cmH_2_O)	21 [12–23]	19 [12–25]	21 [13–22]	0.83
PEEP (cmH_2_O)	12 [10–15]	11 [9–13]	13 [11–15]	0.67
ECMO output (L/min)	4.9 [4.7–5.5]	4.8 [4.7–5.0]	5.0 [4.7–5.6]	0.39
ECMO sweep (L/min)	6.5 [5.0–10.0]	6.3 [5.8–7.4]	6.5 [5.0–10.0]	1.0
ECMO O_2_ fraction (%)	100 [100–100]	90 [80–100]	100 [100–100]	0.07
Lowest PaO_2_ on 100% FiO_2_ in the first 5 days of ECMO (mmHg)	48 [43–52]	45 [42–52]	48 [45–52]	0.55
BAL fluid cells				
Number of cells (10^3^/ml)	45.0 [9.1–256.1]	180.0 [90.2–256.5]	45.0 [11.1–66.6]	0.92
Neutrophils (%)	73 [24–82]	11 [2–34]	77 [60–83]	0.11
Lymphocytes (%)	11 [7–17]	42 [10–75]	8 [5–13]	0.12
Macrophages (%)	14 [7–24]	23 [18–36]	14 [7–28]	0.50
Eosinophils (%)	0 [0–0]	0 [0–0]	0 [0–0]	1.0
Blood cell subpopulation counts				
White blood cells (G/L)	17 [14–32]	18 [15–23]	16 [14–32]	1.0
Neutrophils (%)	79 [76–87]	78 [76–81]	82 [77–88]	0.52
Lymphocytes (%)	7.7 [4.7–8.0]	7.9 [6.8–8]	7.2 [4.2–7.9]	0.59
Macrophages (%)	4.8 [3–6.3]	3.8 [2.7–4.7]	6.15 [3.7–7.0]	0.16
Eosinophils (%)	0.1 [0.0–0.4]	0.05 [0.0–1.1]	0.2 [0.0–0.4]	0.74
CD3+CD4+ T lymphocytes (/μl)	359 [263–605]	575 [474–670]	263 [141–335]	0.11
CD3+CD8+ T lymphocytes (/μl)	221 [119–366]	271 [224–402]	151 [88–341]	0.39
CD3+CD4+/CD3+CD8+ ratio	1.5 [1.2–1.9]	1.8 [1.6–2.1]	1.3 [1.2–1.6]	0.39
NK T lymphocytes (/μl)	124 [72–176]	171 [154–189]	76 [56–106]	0.09
CD19+ B lymphocytes (/μl)	424 [241–782]	680 [447–888]	288 [116–449]	0.20

*Note*: Data are expressed as median [percentiles 25th–75th] or percentages. Comparisons were performed using Mann–Whitney or *χ* ^2^ tests as appropriate.

Abbreviations: BAL, bronchoalveolar lavage; ECMO, extracorporeal membrane oxygenation; NK, natural killer.

Clinical characteristics and biological parameters did not differ between the patients who were weaned from ECMO/ventilator and those who were not (Table 1). No significant differences were found in peripheral white blood cells or lymphocyte subpopulations in relation to successful weaning (Table 2).

**FIGURE 1:**
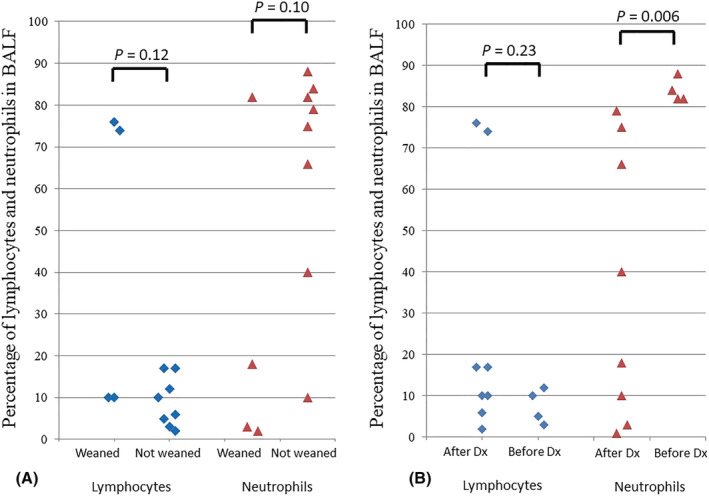
Bronchoalveolar lavage fluid (BALF) lymphocytes and neutrophils in 12 SARS‐Cov2‐related pneumonia patients treated with optimized mechanical ventilation and veno‐venous extracorporeal membrane oxygenation (ECMO) according to their successful weaning from (A) ECMO and ventilator and (B) dexamethasone (DXM) administration. Black lozenges represent lymphocyte proportion and gray triangles represent neutrophil proportion in BALF

Lymphocyte proportion among BAL cells did not differ between the patients weaned from ECMO/ventilator and those not weaned (42% [10–75] vs. 8% [5–13], *P* = 0.12) nor did neutrophils (11% [2–34] vs. 77% [60–83], *P* = 0.11; Figure 1A).

Interestingly, BAL neutrophil proportion was significantly higher in the dexamethasone‐free patients than in the patients with BAL performed after dexamethasone initiation (83% [82–85] vs. 29% [8–68], *P* = 0.006) while lymphocytes represented 8% [5–11] vs. 14% [9–31] of BAL cells, respectively (*P* = 0.23; Figure 1B). No significant differences were found in peripheral white blood cell or lymphocyte subpopulations in dexamethasone‐free patients versus those who received dexamethasone before BAL.

## 4 DISCUSSION

One major finding in this exploratory study is that BAL with >50% neutrophil count was not necessarily associated with evidence on BAL of bacterial superinfection in severe COVID‐19 patients despite the high prevalence of pulmonary infections in ICU COVID‐19 patients including those on ECMO,^11^, ^12^, ^13^ and may thus just be the effect of the viral infection itself. This is important, as in patients with high BAL neutrophil count clinicians may strongly suspect bacterial superinfection and delay steroid initiation. Previously, BAL specimens mainly showed marked lymphocytosis with activated plasma cells.^6^, ^7^

Another important finding was that patients who received dexamethasone before BAL showed significantly lower BAL neutrophil proportions than dexamethasone‐free patients, suggesting that dexamethasone might limit neutrophil recruitment. Whether this effect may contribute to dexamethasone‐induced improvement in survival^3^ remains to be established. Altogether, our results may have implications regarding the immunomodulatory therapy effects if confirmed in future studies. Nevertheless, as expected, the cytological pattern of BAL in our study did not provide predictive information on the possible successful weaning from ECMO/ventilator, which is a multifactorial outcome not easy to predict on the first days of ECMO.

Limitations of our study include the single‐center setting and the relatively small number of patients, which may have precluded evidencing significant differences in BAL neutrophil percentage between weaned and nonweaned patients or identifying plasma IL‐6 thresholds categorizing patient severity, as previously reported.^14^ Therefore, larger cohort studies are necessary to better explore these aspects. However, to the best of our knowledge, this is the first study to investigate BAL cells in ECMO‐treated COVID‐19 patients; a single case has been previously published.^6^ Logistical difficulties related to the pandemic precluded a more complex design.

In conclusion, in this exploratory study, high neutrophil proportion in BALF can be observed in severely SARS‐Cov2‐infected patients with no microbiological evidence on BAL of bacterial superinfection at the time of BAL. Dexamethasone administration is associated with a decrease in BAL neutrophil proportion, a finding that needs further investigation.

## CONFLICT OF INTEREST

None declared.

## AUTHOR CONTRIBUTIONS

SV and BM conceived of and designed the study. SV, IM, APL, LS, GN, AM, EG, JME, ND, and BM took care of the patients, implemented the ECMO, and performed the bronchoalveolar lavage. HAB analyzed the bronchoalveolar lavage fluid. All authors acquired, analyzed, and interpreted the data. SV and BM drafted the manuscript. All authors participated to the critical revision of the manuscript for important intellectual content.

## ETHICS STATEMENT

The study was part of the COVID‐ICU and French COVID‐19 cohort registries and approved by our institutional ethics committee (IDRCB, 2020‐A00256‐33; CPP, 11‐2020.02.04.68737). All patients or next of kin gave informed consent to participate.

## Data Availability

BM has full access to all data and takes responsibility for the data integrity and its analysis accuracy.
